# The flagella of ‘*Candidatus* Liberibacter asiaticus’ and its movement *in planta*

**DOI:** 10.1111/mpp.12884

**Published:** 2019-11-13

**Authors:** Maxuel O. Andrade, Zhiqian Pang, Diann S. Achor, Han Wang, Tingshan Yao, Burton H. Singer, Nian Wang

**Affiliations:** ^1^ Citrus Research and Education Center Department of Microbiology and Cell Science University of Florida/Institute of Food and Agricultural Sciences Lake Alfred FL USA; ^2^ National Engineering Research Center for Citrus, Citrus Research Institute, Southwest University Chongqing 400712 People’s Republic of China; ^3^ Emerging Pathogens Institute University of Florida Gainesville FL USA

**Keywords:** citrus, flagella, HLB control, huanglongbing, Liberibacter, movement, psyllid

## Abstract

Citrus huanglongbing (HLB) is the most devastating citrus disease worldwide. ‘*Candidatus* Liberibacter asiaticus’ (Las) is the most prevalent HLB causal agent that is yet to be cultured. Here, we analysed the flagellar genes of Las and *Rhizobiaceae* and observed two characteristics unique to the flagellar proteins of Las: (i) a shorter primary structure of the rod capping protein FlgJ than other *Rhizobiaceae* bacteria and (ii) Las contains only one flagellin‐encoding gene *flaA* (CLIBASIA_02090), whereas other *Rhizobiaceae* species carry at least three flagellin‐encoding genes. Only *flgJ_Atu_* but not *flgJ_Las_* restored the swimming motility of *Agrobacterium tumefaciens flgJ* mutant. Pull‐down assays demonstrated that FlgJ_Las_ interacts with FlgB but not with FliE. Ectopic expression of *flaA_Las_* in *A. tumefaciens* mutants restored the swimming motility of ∆*flaA* mutant and ∆*flaAD* mutant, but not that of the null mutant ∆*flaABCD*. No flagellum was observed for Las in citrus and dodder. The expression of flagellar genes was higher in psyllids than *in planta*. In addition, western blotting using flagellin‐specific antibody indicates that Las expresses flagellin protein in psyllids, but not *in planta*. The flagellar features of Las *in planta* suggest that Las movement in the phloem is not mediated by flagella. We also characterized the movement of Las after psyllid transmission into young flush. Our data support a model that Las remains inside young flush after psyllid transmission and before the flush matures. The delayed movement of Las out of young flush after psyllid transmission provides opportunities for targeted treatment of young flush for HLB control.

## Introduction

Citrus huanglongbing (HLB, also called citrus greening) is the most devastating citrus disease worldwide. The most prevailing HLB pathogen in the world is ‘*Candidatus* Liberibacter asiaticus’ (Las), an unculturable bacterium vectored by Asian citrus psyllid (ACP, *Diaphorina citri*), which is an invasive pest for citrus‐producing areas outside of Asia (Bové, 2006; Wang and Trivedi, 2013). Currently, HLB management strategies mainly rely on psyllid control and numerous horticultural approaches (Blaustein *et al.*, 2018; Li *et al.*, 2016, 2017, 2018) since all commercial citrus varieties are susceptible to HLB (Folimonova *et al.*, 2009). Current HLB and ACP management has not prevented HLB from spreading worldwide (Wang, 2019). For example, HLB has spread throughout Florida, to other major citrus‐producing states (e.g. California and Texas) in the USA, and to neighbouring countries including Mexico and countries in the Caribbean and Central America (Wang *et al.*, 2017). It is paramount to understand the biology and virulence mechanism of Las to design a suitable and efficient HLB control strategy. However, its unculturability has hampered our investigation of the biology and virulence mechanism of Las. Its close relatives, such as *Liberibacter crescens*, *Agrobacterium* and *Sinorhizobium*, have been used as surrogates in previous studies (Andrade and Wang, 2019; Jain *et al.*, 2019; Naranjo *et al.*, 2019; Vahling‐Armstrong *et al.*, 2012).

**Figure 1:**
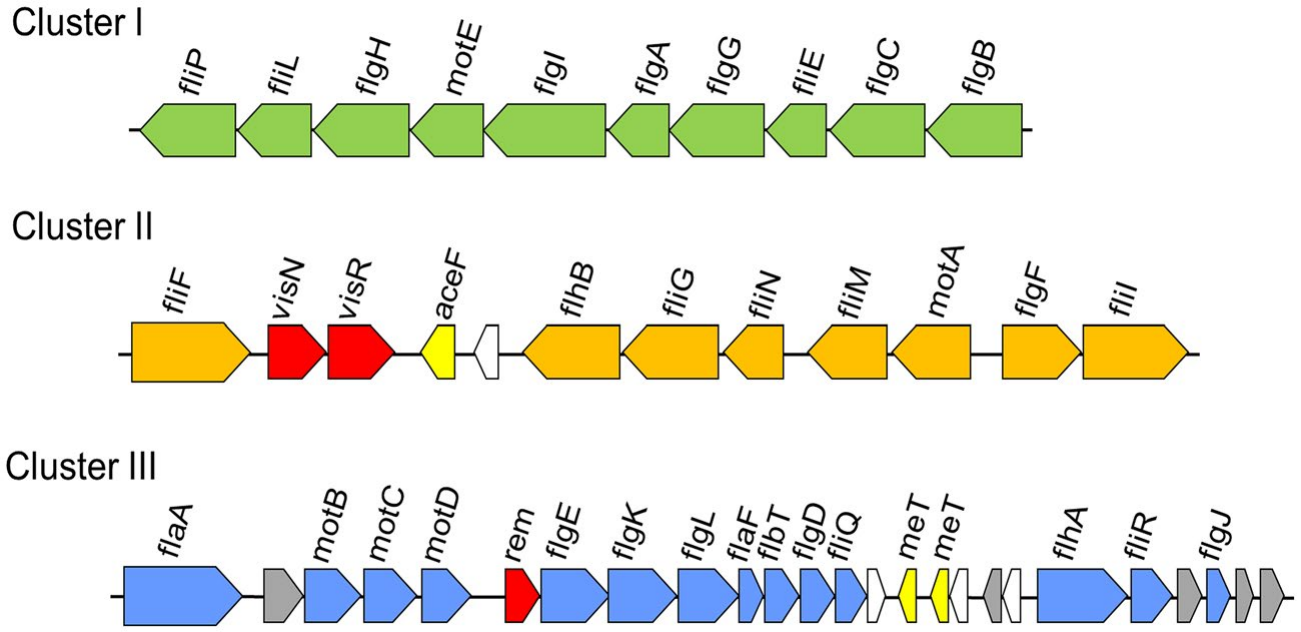
Genetic organization of the three clusters encoding flagellar genes in the ‘*Candidatus* Liberibacter asiaticus’ genome. The names of the coding regions shown in the diagram are based on their homology with flagellar genes in *Rhizob* *i* *aceae* bacteria. Grey arrows, conserved hypothetical protein; white arrows, hypothetical proteins.

Las has been observed to be distributed throughout the whole plant, including leaf, stem, root, flower and seed coat (Tatineni *et al.*, 2008). However, the details of Las movement *in planta* are still unknown. Most bacteria possess more than one organelle for motility. For most bacteria, flagella are the major motility organelles responsible for swimming and swarming, whereas type IV pili are responsible for the twitching motility. Las encodes the type IVc tight adherence‐pili (Tad) that are usually not associated directly with motility. Instead, they are involved in adherence (Andrade and Wang, 2019). Flagellum‐mediated motility allows bacterial cells to move toward and explore nutrient‐rich habitats and move away from unfavourable environments (Anderson *et al.*, 2010; Berry and Armitage, 1999; Moens and Vanderleyden, 1996; Zhu *et al.*, 2013). Flagella also play a key role in surface attachment and host–bacteria interactions (Heindl *et al.*, 2014; Rossez *et al.*, 2015). It has been known that some flagellated plant pathogenic bacteria suppress expression of flagella in the plant, probably to avoid triggering plant defence (Chatnaparat *et al.*, 2016; Yu *et al.*, 2013). Las contains 30 flagellar genes located in three clusters on the chromosome (Fig. 1) (Duan *et al.*, 2009). However, it remains to be determined whether Las synthesizes flagella during within‐plant movement. This is a fundamental question that has clear implications for the rate at which disease symptoms appear in both new flush and old leaves.

The flagellar machinery can be divided into five parts called the basal body, the hook, the hook–filament junction zone, the filament and the filament cap (DeRosier, 2006; Evans *et al.*, 2014; Ghosh, 2004; Minamino and Namba, 2004). The basal body is composed of the cytoplasmatic C‐ring (FliG, FliM and FliN), the inner or MS‐ring (FliF), the periplasmatic P‐ring (FlgI), the outer membrane L‐ring (FlgH), the proximal rod (FlgB, FlgC, FlgF and FliE) and the distal rod (FlgG) (Jones and Aizawa, 1991; Park *et al.*, 2006). The basal body is embedded in the cell surface and plays a role in flagella rotation together with the Mot proteins (Minamino *et al.*, 2008). The hook and filament, which are tubular structures composed of subunits of hook (FlgE) and flagellin (FliC), respectively, extend outwards from the cell (Macnab, 2003). The hook length and the switch of specificity from substrates hook‐to‐filament are tightly regulated by FliK (Erhardt *et al.*, 2010; Waters *et al.*, 2007). Deletion of *fliK* leads to formation of prolonged flagellar hooks (polyhooks), lack of filament structures and nonmotile phenotypes (Minamino *et al.*, 2009). The hook–filament junction zone (FlgK and FlgL) and the filament cap (FliD) are located between the hook and filament and at the tip of the filament, respectively (Ikeda *et al.*, 1996). Flagellar assembly begins with the basal body, proceeds with the hook and finishes with the filament. Capping proteins (FlgJ, FlgD and FliD) are needed to permit and regulate the polymerization of rod, hook and filament, respectively, at different stages of flagellar assembly (Minamino and Namba, 2004). FlgJ, FlgD and FliD exist at the tip of the growing rod, hook and filament structures, respectively (Evans *et al.*, 2014).

The flagellar export apparatus is built into the central pore of the basal body MS‐ring, and it is formed by interaction of six membrane proteins: FlhA, FlhB, FliP, FliO, FliQ and FliR, and three cytoplasmic proteins FliH, FliI and FliJ. FliI is the ATPase that provides the energy for the translocation of proteins across the cytoplasmic membrane (Macnab, 2003). FliH acts as an ATPase regulator, coupling the energy of ATP hydrolysis to flagellar protein export (Evans *et al.*, 2014; Macnab, 2003). Also, the chaperone, FliJ, prevents premature aggregation of substrates in the cytoplasm, and FlgA, assists the P‐ring assembly in the periplasm (Evans *et al.*, 2006; Macnab, 2003).

Flagellins are the subunits that compose the flagellar filament and represent the major flagellar structural protein (Altegoer *et al.*, 2014; Rossez *et al.*, 2015). The number of flagellin‐encoding genes in bacterial genomes varies and may range between one (*Escherichia coli*) and seven (*Vibrio parahaemolyticus*) (Fedorov and Kostyukova, 1984; Kim and McCarter, 2000; Stewart and McCarter, 2003). The flagellar filaments in *E. coli* K‐12 are built up from only one flagellin protein (FliC) (Turner *et al.*, 2012), whereas *Bdellovibrio bacteriovorus* (Thomashow and Rittenberg, 1985), *Campylobacter coli* (Guerry *et al.*, 1990), *Caulobacter crescentus* (Driks *et al.*, 1989), *Agrobacterium tumefaciens* (Deakin *et al.*, 1999; Mohari *et al*., 2018), *Rhizobium lupini* strain H13‐3 and *Sinorhizobium meliloti* have at least two flagellin proteins forming their flagellar filaments (Scharf *et al.*, 2001).

Young flush has been shown to be critical for ACP and Las infection. ACP preferentially feeds and exclusively reproduces on young, newly emerged flush shoots of citrus. ACP nymphs feed and complete their life stages on young flush shoots. This observation appears to be universal across different citrus‐producing regions worldwide (Bové, 2006; Hall *et al.*, 2013; Setamou *et al.*, 2016; Sétamou and Bartels, 2015; Tomaseto *et al.*, 2016). Not surprisingly, citrus trees are normally infected when new flush is present (Hall *et al.*, 2016). In addition, ACPs that acquire Las as adults are poor vectors of Las compared with ACPs that acquire the pathogen as nymphs (Pelz‐Stelinski *et al.*, 2010). A threshold infection level (*c*. 10^6^ Las/psyllid) is required for successful transmission to citrus plants (Ukuda‐Hosokawa *et al.*, 2015). How Las moves after psyllid transmission remains largely unexplored.

Here, we investigated the flagella of Las. Our results indicate that Las flagellar genes express at low levels *in planta*. Las moves to other sink tissues with phloem sap when young leaves mature into sources. This delayed Las movement after ACP transmission into young flush might present opportunities to develop new strategies for HLB management.

## Results

### Comparison of flagellar genes of ‘*Ca.* Liberibacter’ and other members of *Rhizobiacea* *e*

The analysis of the flagellar components encoded by the Las genome (Fig. 1 and Table 1) showed the following characteristics that are shared by all bacterial species in *Rhizobiaceae*: (i) lack of cytoplasmic chaperone FliJ and the components of export apparatus FliH/FliO; (ii) the function of FliK is performed by MotD (Eggenhofer *et al.*, 2006); (iii) the rod‐capping FlgJ protein lacks the muramidase domain in its carboxy‐terminus (Herlihey *et al.*, 2014); and (iv) lack of FliD, the filament cap (Table 1). It is important to note that all the traits cited above are shared by members of *Rhizobiaceae* that are known to contain flagellar apparatus and show swimming behaviour, such as *A. tumefaciens* and *S. meliloti* (Attmannspacher *et al.*, 2005; Deakin *et al.*, 1999; Mohari et al., 2018).

**Table 1:** Identity of residues of flagellar proteins encoded by *Rhizobiaceae* bacteria (as percentage values)

Gene	Las	Atu	Sme	Lcc	Laf	Lso
*Basal body*						
FliG	**+**	46	45	54	81	82
FliM	+	30	30	42	70	66
FliN	+	47	50	50	77	74
FliF	+	49	50	56	78	75
FlgI	+	64	64	68	85	87
FlgH	+	54	56	63	80	76
FlgB	+	45	47	57	76	75
FlgC	+	59	62	68	79	85
FlgF	+	44	46	49	77	75
FliE	+	37	37	42	67	63
FlgG	+	59	62	69	88	90
FlgJ	+*	29	32	37*	61*	68*
MotA	+	62	58	64	60	63
MotB	+	31	32	36	65	75
MotC	+	29	27	34	65	60
*Hook*						
FlgE	+	50	41	58	75	74
FliK/MotE	+	32	28	29	44	48
FlgD	+	43	44	57	85	75
*Junction zone*						
FlgK	+	36	36	47	76	69
FlgL	+	27	30	39	61	57
*Filament*						
FlaA	+	50	42	49	67	61
FlaB	†	+	+	−	−	−
FlaC	†	+	+	−	−	−
FlaD	†	+	+	−	−	−
*Capping filament*						
FliD	†	†	†	†	†	†
*Export apparatus*						
FlhA	+	66	65	68	86	82
FlhB	+	44	45	54	80	74
FliP	+	60	57	67	90	86
FliO	†	†	†	†	†	†
FliQ	+	57	61	65	90	90
FliR	+	40	43	51	81	78
FliH	†	†	†	†	†	†
FliI	+	62	63	68	87	84
*Chaperones*						
FliJ	†	†	†	†	†	†
FlgA	+	42	46	53	70	69

Note:

Comparative analysis of flagellar proteins encoded by different species of *R* *h* *izob* *i* *aceae* bacteria. Las, ‘*Candidatus* Liberibacter asiaticus’ (CP001677); Laf, ‘Candidatus Liberibacter africanus’ (CP004021); Lso, ‘*Candidatus *Liberibacter solanacearum’ (CP002371); Lcc, *Liberibacter crescens* strain BT‐1 (CP003789); Atu, *Agrobacterium tumefaciens* (AE007869); Sme, *Sinorhizobium meliloti* strain 1041 (AL591688).

* Indicates a shorter primary structure for FlgJ homologues when compared to *A. tumefaciens* and *S. meliloti*.

† No homologues were identified. + and − indicate presence and absence of a homologue in the analysed genome, respectively.

**Figure 2:**
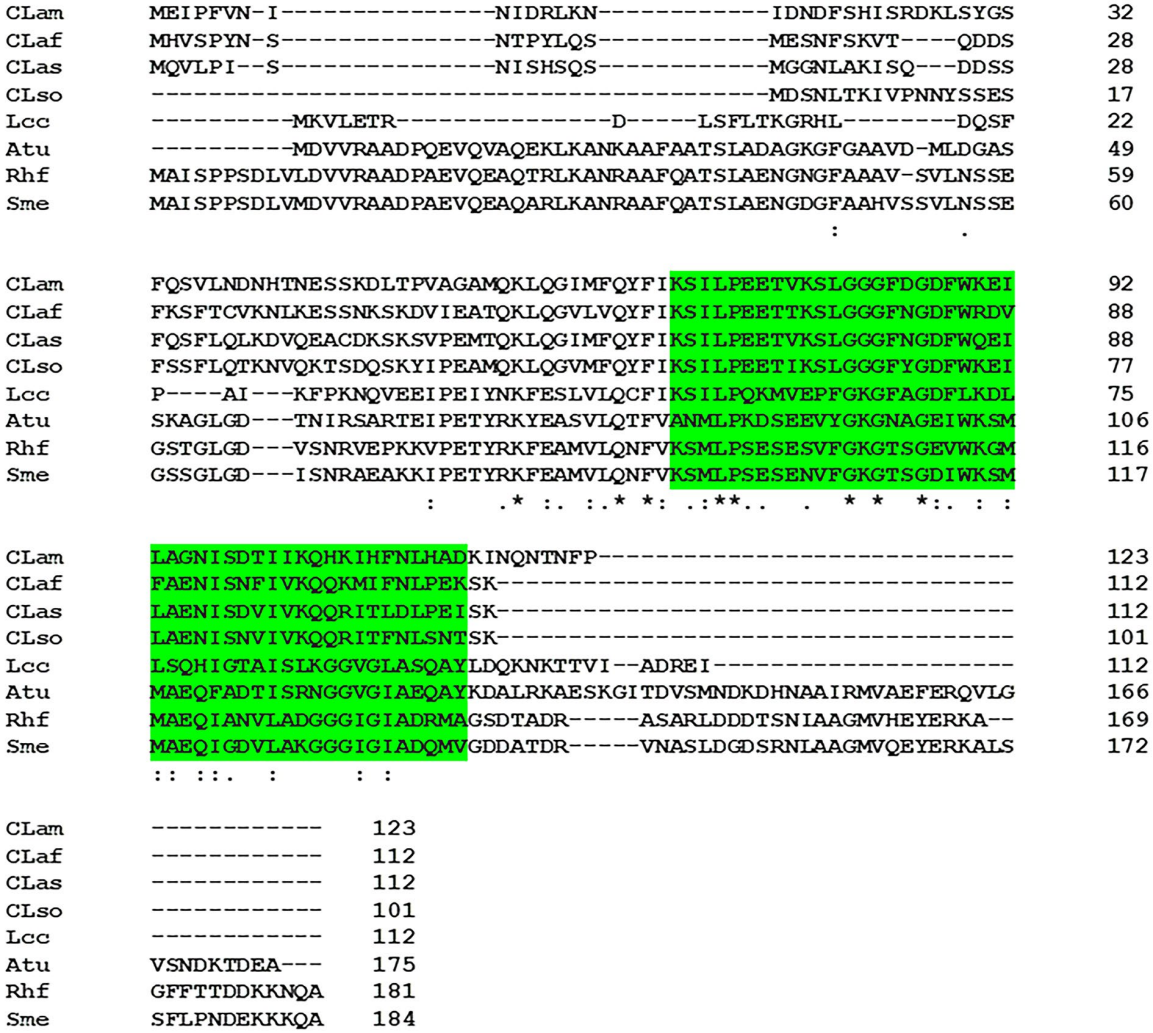
Sequence alignment of the rod cap proteins (FlgJ) encoded by *Rhizob* *i* *aceae* bacteria showing the rod‐binding domain highlighted in green. Atu, *Agrobacterium tumefaciens* (AE007869); Sme, *Sinorhizobium meliloti* strain 1041 (AL591688); Rhf, *Sinorhizobium fredii* NGR234 (CP001389); CLas, ‘*Candidatus* Liberibacter asiaticus’ (CP001677); CLaf, ‘*Candidatus* Liberibacter africanus’ (CP004021); CLso, ‘*Candidatus* Liberibacter solanacearum’ (CP002371); Lcc, *Liberibacter crescens* strain BT‐1 (CP003789); CLam, ‘*Candidatus* Liberibacter americanus’ (CP006604). Alignment result was obtained by ClustalW and protein domain was identified with Pfam.

We observed two characteristics unique to the flagellar proteins of Las and *Liberibacter crescens* BT‐1, a culturable bacterium, that may determine the fate of the Las flagellar machinery:

1. It has a shorter primary structure of the rod‐capping protein FlgJ than other *Rhizobiaceae* bacteria (Fig. 2). In previous work, it was shown that the minimal functional region required of FlgJ for promoting the rod assembly consists of 151 residues in its amino terminus (Hirano *et al.*, 2001). The primary structure of FlgJ in Las (CLIBASIA_01980) contains 112 amino acid residues, whereas in *A. tumefaciens* (Atu0584) and *S. meliloti* (SMc03071), FlgJ homologues consist of 175 and 184 residues, respectively.
2. Las contains only one flagellin‐encoding gene *flaA* (CLIBASIA_02090), whereas other *Rhizobiaceae* species carry at least three flagellin‐encoding genes (Fig. 1, Table 1). For example, *Rhizobium lupini* H13‐3 and *S. meliloti* produce flagellar filaments composed of three and four flagellin subunits, respectively (Scharf *et al.*, 2001). These flagellin‐encoding genes are named *flaA* through *flaD.* Indeed, the complex flagellar filaments in *Rhizobiaceae* bacteria are composed of a majority flagellin and at least one secondary flagellin. Mutational analysis of flagellin genes revealed that, in both *R. lupini* and *S. meliloti*, FlaA is the principal flagellin and that FlaB, FlaC and FlaD are secondary (Deakin *et al.*, 1999). In this way, FlaA and at least one secondary flagellin are required for assembling a functional flagellar filament in *R. lupini* and *S. meliloti* (Scharf *et al.*, 2001). Similar results were demonstrated in *A. tumefaciens* (Mohari et al., 2018). Thus, the unique features of Las in FlgJ and FlaA are probably critical to functionality of its flagella.

### Characterization of the function of FlgJ and FlaA of Las using *Agrobacterium* as a surrogate

**Figure 3:**
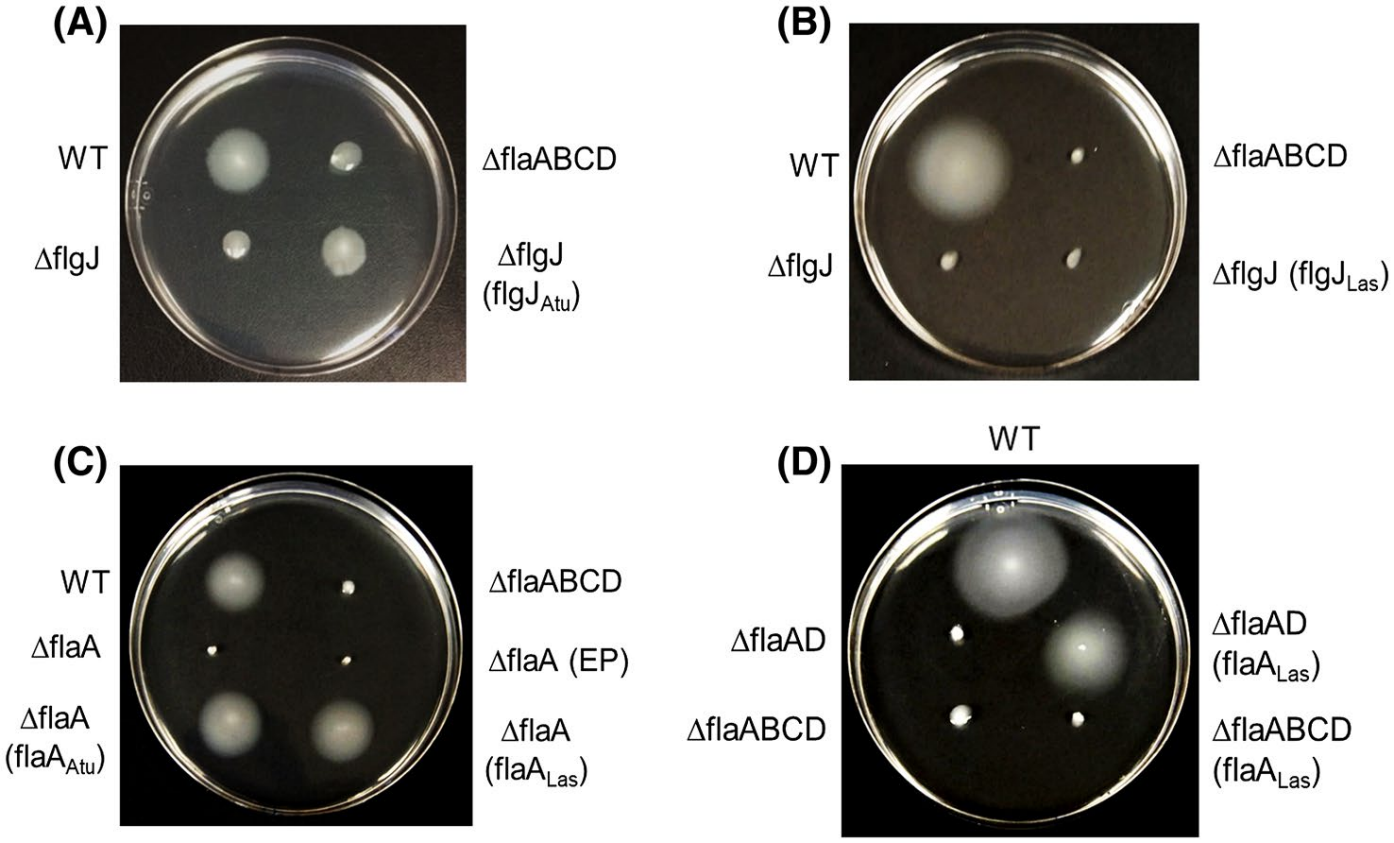
Functional analysis of FlgJ and FlaA of ‘*Candidatus* Liberibacter asiaticus’ (Las) via complementation analysis of their corresponding mutants of *Agrobacterium tumefaciens* (Atu) with swimming assay. (A) Complementation of *A. tumefaciens flgJ* mutant with *flgJ_Atu._*. (B) Complementation of *A. tumefaciens flgJ* mutant with *flgJ_Las_*. (C) Complementation of *A. tumefaciens flaA* mutant with *flaA_Las_* and *flaA_Atu_*. (D) Complementation of *A. tumefaciens flaAD* and *flaABCD* mutants with *flaA_Las_ .* WT**,** *A. tumefaciens* strain C58; ∆*flaABCD*, flagellum null mutant; ∆*flgJ*, *A. tumefaciens* C58 with deletion of *flgJ*; ∆*flaA*, *A. tumefaciens* C58 with in‐frame deletion in *flaA*; ∆*flgJ* (*flgJ_Atu_*), *flgJ* mutant complemented with *A. tumefaciens flgJ*; ∆*flgJ* (*flgJ_Las_* ), *flgJ* mutant complemented with Las *flgJ*; ∆*flaA* (*flaA_Atu_*), *flaA* mutant complemented with *A. tumefaciens flaA*; ∆*flaA* (*flaA_Las_*), *flaA* mutant complemented with Las *flaA*; ∆*flaA (EP)*, ∆*flaA* carrying empty plasmid; ∆*flaAD*, *A. tumefaciens* C58 with in‐frame deletion for the flagellin‐encoding genes *flaA* and *flaD*; ∆*flaABCD*, *A. tumefaciens* C58 carrying deletion for all flagellin genes *flaA*, *flaB*, *flaC* and *flaD*; ∆*flaAD* (*flaA_Las_*) and ∆*flaABCD* (*flaA_Las_*), mutants ∆*flaAD* and ∆*flaABCD* harbouring the flagellin‐encoding gene *flaA* from Las. Bacterial cells were incubated at 28 °C and photographed after 3 days.

**Figure 4:**
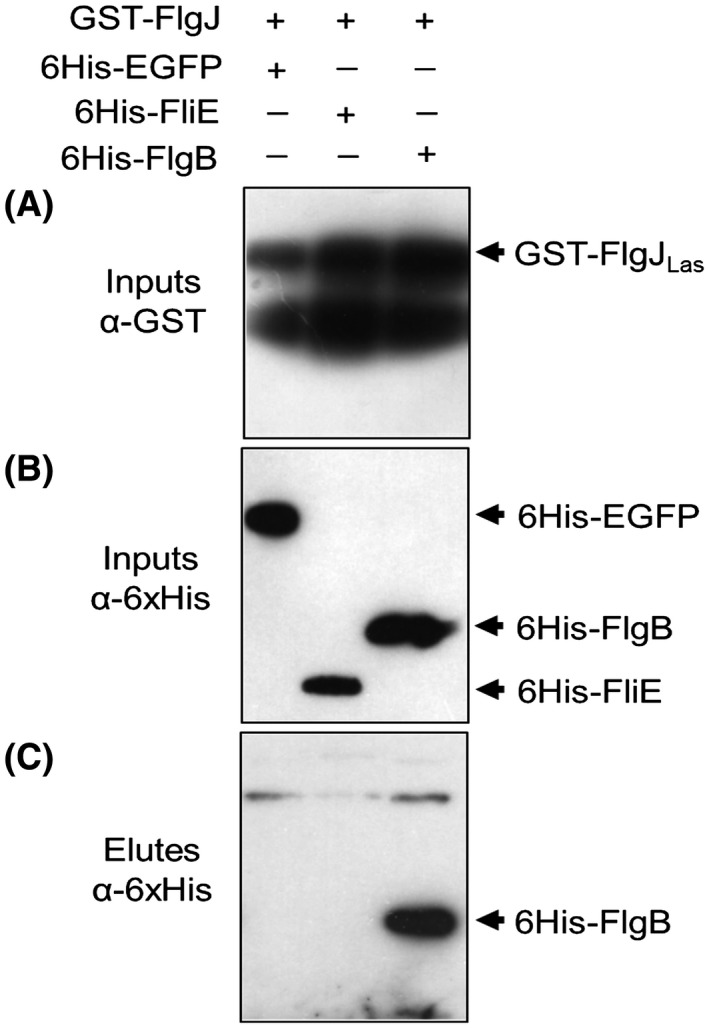
Pull‐down assay showed a physical interaction between FlgJ_Las_ and FlgB. (A) The protein GST‐FlgJ_Las_ was used as a bait for the pull‐down assay. GST‐FlgJ_Las_ was expressed in *Escherichia coli*, immobilized and washed on glutathione sepharose beads, and incubated with *E. coli* lysates containing 6×HisEGFP, 6×HisFlgB or 6×HisFliE.  Pull‐down inputs containing the supernant of total cell extracts (A) and (B) were immunobloted using the anti‐GST and anti‐6×His antibodies, respectively. Eluted protein fractions (Elutes) were probed with anti‐6×His antibodies.

We first evaluated whether FlgJ and FlaA of Las are functional by assessing whether they can complement corresponding mutants of *A. tumefaciens* (Atu). For this purpose, we constructed a nonpolar mutant of *flgJ* in *A. tumefaciens.* As expected, the *flgJ* mutant of *A. tumefaciens* became immobile, which is similar to the ∆*flaABCD* mutant with all four flagellin genes deleted (Fig. 3). Only *flgJ_Atu_* but not *flgJ_Las_* restored the swimming motility of *A. tumefaciens flgJ* mutant (Fig. 3A,B), indicating *flgJ_Las_* might not be functional or works differently in Las. To verify whether FlgJ_Las_ can physically interact with the proximal rod proteins FlgB and FliE as shown in other well‐studied systems (Hirano *et al.*, 2001), we performed a pull‐down experiment with those proteins. Interestingly, FlgJ_Las_ interacted with FlgB but not with FliE (Fig. 4).

To test the function of flagellin protein FlaA_Las_, we generated mutant strains of *A. tumefaciens* carrying deletion in *flaA* alone (∆*flaA*), in *flaA* and *flaD* genes (∆*flaAD*), and in all flagellin‐encoding genes (∆*flaABCD*)*.* Interestingly, ectopic expression of *flaA_Las_* in *A. tumefaciens* mutants restored the swimming motility of ∆*flaA* mutant (Fig. 3C) and ∆*flaAD* mutant (Fig. 3D), but not that of the null mutant ∆*flaABCD* (Fig. 3D). The swimming halos for the tested strains were calculated and the means and standard deviation (*n* = 3) values are shown in Fig. S2. These results indicate that in the *A*. *tumefaciens* flagellar system FlaA_Las _functions as the majority flagellin interacting with other secondary flagellins such as FlaB or FlaC, which remain intact in the ∆*flaAD* mutant, to form a fully functional flagellar filament. However, FlaA_Las_ alone could not form a fully functional flagellar filament in *A. tumefaciens*.

#### Observation of Las flagellum in psyllids and *in planta*

**Figure 5:**
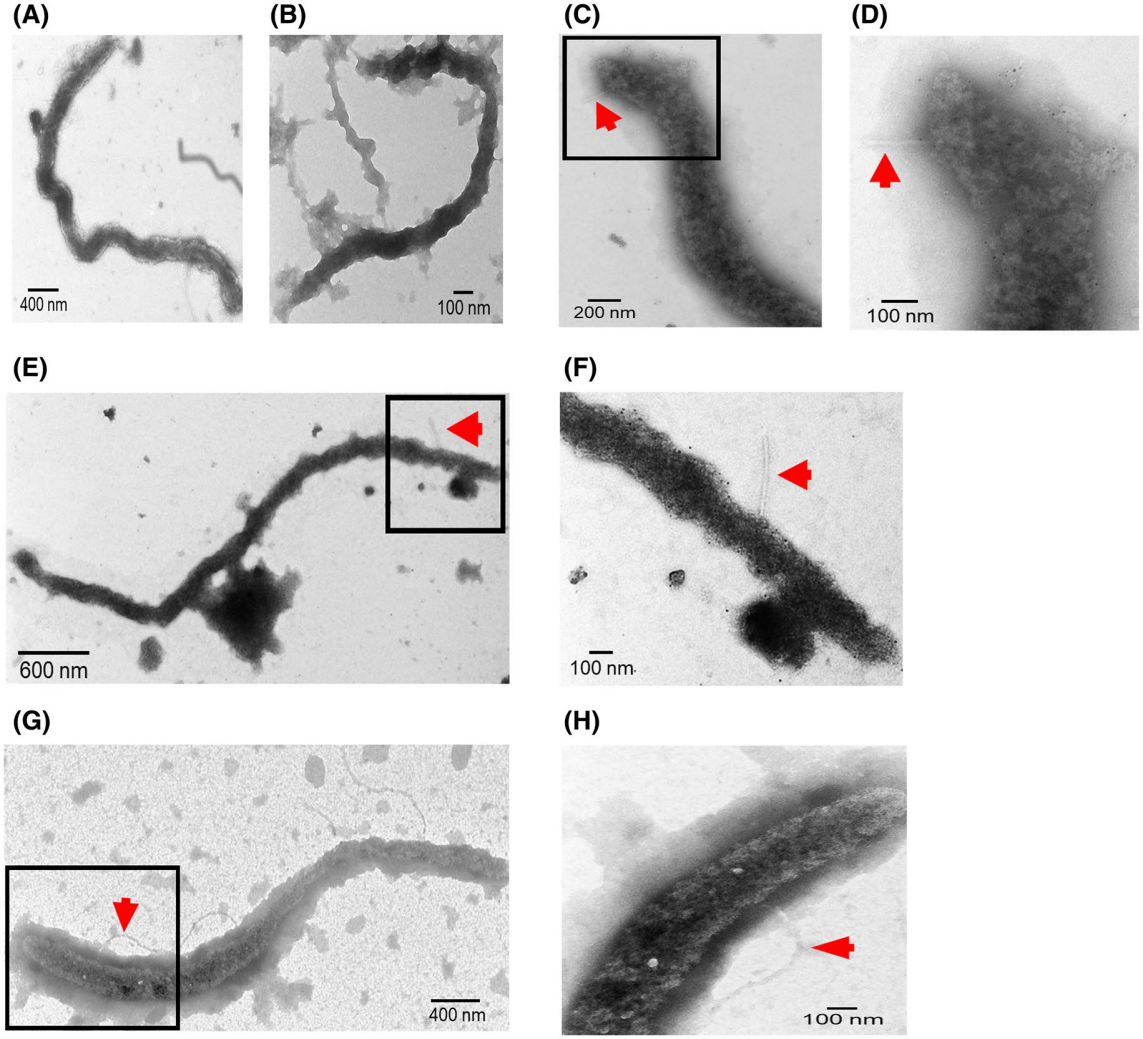
Transmission electron microscopy analysis of ‘*Candidatus* Liberibacter asiaticus’ (Las). (A) Las cell isolated from Las‐infected grapefruit seed coat. (B) Las cell isolated from dodder stem. (C)–(H) Las isolated from psyllid guts. (D), (F) and (H) are enlarged views of the indicated area in (C), (E) and (G), respectively. Las cells were visualized by using negative staining and antibody against Las OmpA and second antibody anti‐rabbit IgG‐gold. Arrows indicate thread‐like structures. Size bars are indicated in the images.

**Figure 6:**
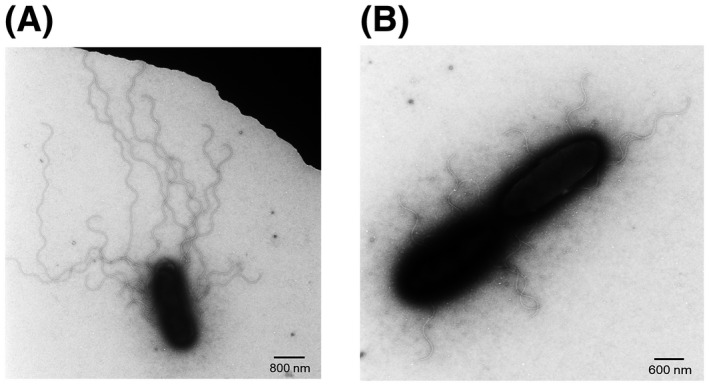
Visualization of the flagellar structures in *Liberibacter crescens* BT‐1 cells. Negatively stained cells were analysed by transmission electron microscopy. Size bars are indicated in the images.

To verify whether Las forms a functional flagellum, we visualized Las cells isolated from Las‐infected plants and psyllids using transmission electron microscopy (TEM). For the visualization, Las cells were confirmed using Las‐specific antibody against Las OmpA and the second antibody anti‐rabbit IgG‐gold. Las cells isolated from grapefruit (*Citrus paradisi*) seed coats (Fig. 5A) and parasitic dodder stems (*Cuscuta* sp.) and psyllids are all filament‐shaped or long‐rod‐shaped. Previous observation of Las under TEM suggested that it is polymorphic with Las cells being mostly filamentous in psyllids, but in different shapes in plants with diameter 0.33–1.5 μm and length 2.6–6.3 μm (Hartung *et al.*, 2010). No flagellum was observed for Las cells isolated from grapefruit seed coats (Fig. 5A) and parasitic dodder stems (Fig. 5B). For Las isolated from psyllid guts, no flagellum was observed on most Las cells. A few Las cells showed thread‐like structures from which we could not make a conclusive claim whether they are indeed flagella (Fig. 5C‐H). However, *L. crescens*, which shares the same flagellar genes as Las (Table 1), was observed to contain flagella under TEM (Fig. 6).

#### Expression of flagellar genes in psyllids and *in planta*

**Figure 7:**
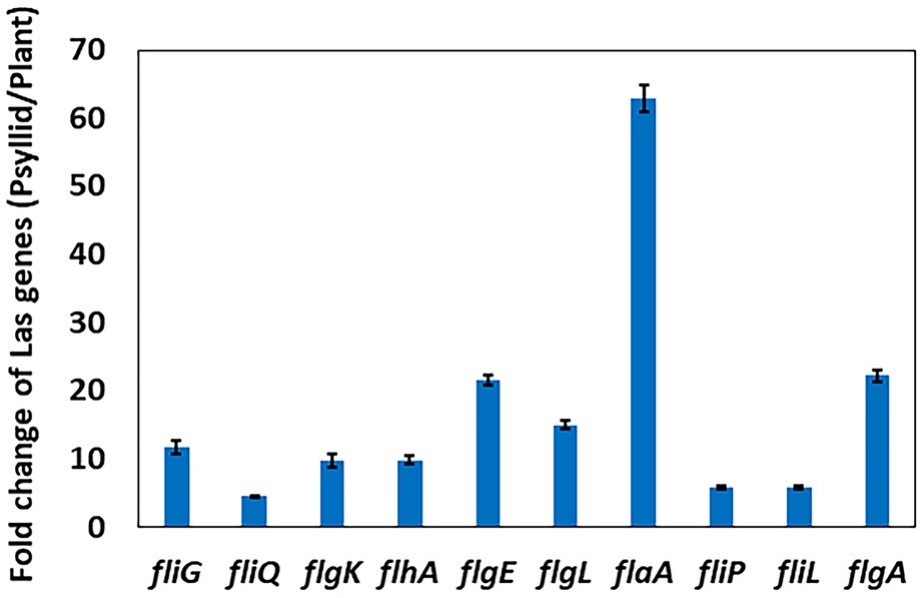
RT‐qPCR analysis of selected ‘*Candidatus* Liberibacter asiaticus’ (Las) genes encoding flagellum apparatus. Fold change is the relative gene expression (in psyllid versus *in planta*) of Las.

**Figure 8:**
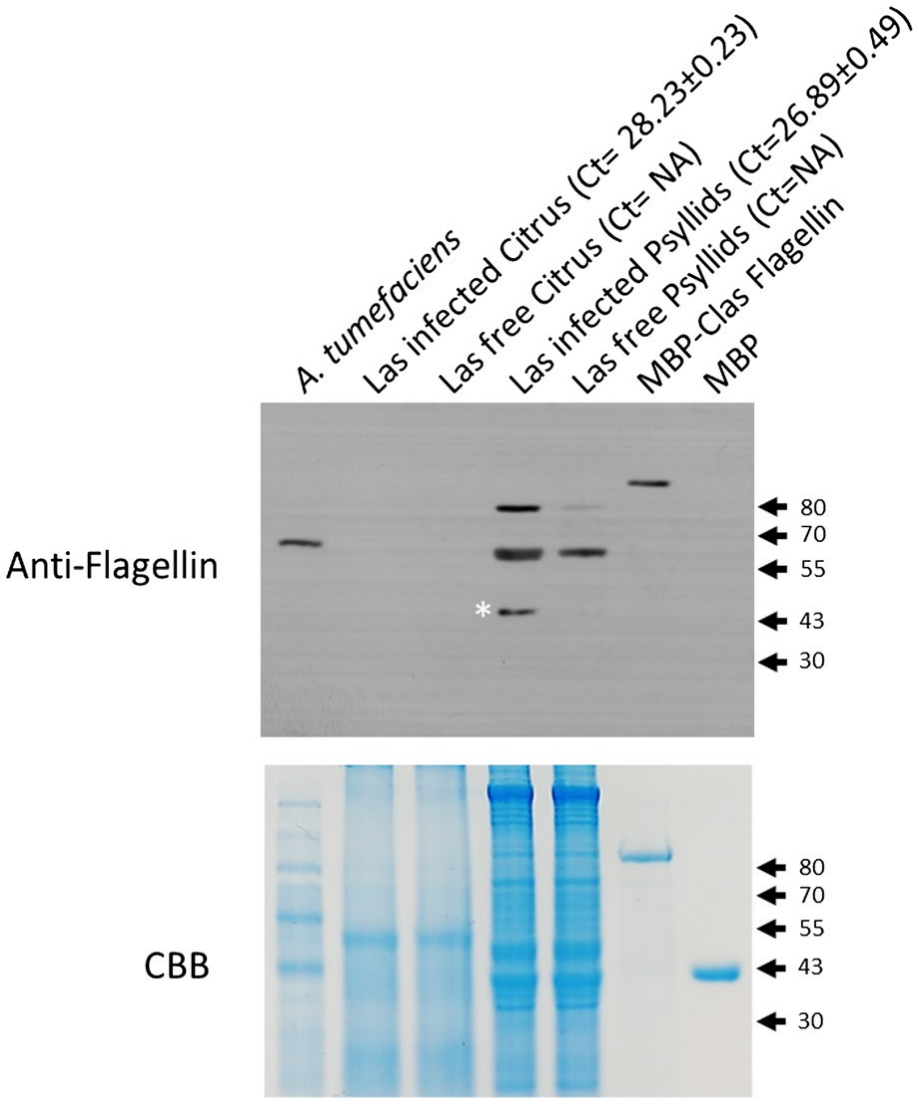
Flagellin detection. The antibody produced using the peptide of ‘*Candidatus* Liberibacter asiaticus’ (Las) flagellin protein (sequence: DRVSSGLRVSDAAD) was used to detect flagellin protein in different bacterial samples (left to right): *Agrobacterium tumefaciens* C58 derivative strain; Las‐infected citrus (Ct = 28.23 ± 0.23); Las‐free citrus control; Las‐infected psyllids (Ct = 26.89 ± 0.49); Las‐free psyllid control; positive MBP‐Las flagellin fusion protein control and negative MBP protein control. The band marked with an asterisk is the flagellin protein of Las with MW 48 kDa. Coomassie brilliant blue (CBB) served as a loading control. The experiment was repeated twice with similar results.

To further assess whether Las synthesizes flagella in psyllids and plants, we tested the expression of flagellar genes at both transcription and translation levels using quantitative reverse transcription PCR (RT‐qPCR). We found that flagellar genes were up‐regulated in psyllids compared to *in planta* (Fig. 7). Specifically, the flagellin gene *flaA* is highly up‐regulated in psyllids, which is consistent with our previous study (Yan *et al.*, 2013). Likewise, flagellin protein was detected for Las inside psyllids, but not inside plants based on western blot (Fig. 8). Taken together, our data suggest Las lacks flagella *in planta* or flagellar genes express at low level *in planta*.

#### Movement of Las *in planta* after ACP transmission of Las into young flush

Las has been known to translocate through the sieve pore to move from the infection sites to tissues throughout the plants (Kim *et al.*, 2009). Since Las does not produce flagella or its flagellar gene expression is low *in planta*, its movement to other parts (e.g. roots) of the plant from the infection site is unlikely to be mediated by flagella. Instead, we hypothesize that Las moves with phloem sap to sink tissues when young flush matures. To test this hypothesis, we caged ten Las‐positive ACPs/flush (Valencia sweet orange) for each plant for different durations and tested for Las at 54 days post‐psyllid feeding. For each treatment, we used five plants with one plant as a biological replicate. After 54 days, 40%, 100%, 100% and 80% of tested leaves were Las‐positive following 6, 11, 18 and 29 days of ACP feeding, respectively. However, none of the root samples were Las‐positive, indicating that Las had not moved to roots.

To further understand Las movement inside plants after ACP transmission, we trimmed nine citrus plants (Hamlin sweet orange on Cleo) to trigger young flush. We caged 15 psyllids/plant together with young flush and five mature leaves. Las was detected in young flush‐developed leaves in five of the nine plants at 4 weeks after ACP transmission, and 7, 9 and 15 weeks after ACP transmission. Las was detected in the roots in five of the infected plants at 15 weeks after ACP transmission, but not at other time points. Las was not detected in all the old leaf samples inside or outside of the cage at the aforementioned time points. Taken together, our data are consistent with our hypothesis that Las moves from source to sink passively with phloem sap and will likely stay in the young flush after ACP transmission and before flush matures.

## Discussion

Las flagella have multiple important features. First, FlgJ of Las is not able to complement the *flgJ* mutant of *A. tumefaciens.* FlgJ plays an important role in flagellar assembly (Hirano *et al.*, 2001). FlgJ_Las,_ as its homologues in several other bacterial phyla, including Alphaproteobacteria, Deltaproteobacteria, Epsilonproteobacteria and Spirochaetes (Nambu *et al.*, 2006), contains only the rod‐capping domain involved in protein–protein interactions, but lacks the peptidoglycan hydrolase domain, which allows the elongating flagellar rod to penetrate through the peptidoglycan (PG) layer. The peptidoglycan hydrolase domain is commonly found in FlgJ from other bacteria but its function can be developed by other peptidoglycan hydrolase protein not related to flagellar proteins. *Liberibacter* genomes code for one peptidoglycan hydrolase, which contains a soluble lytic transglycosylase (SLT) domain and a periplasmic signal peptide (Fig. S1). In enteric bacteria such as *Salmonella enterica* serovar Typhimurium, *flgJ* null mutants fail to produce the flagellar rods, hooks and filaments, and are nonmotile (Hirano *et al.*, 2001; Nambu *et al.*, 1999). Instead, the *flgJ* mutant of *Borrelia burgdorferi*, which contains a single‐domain FlgJ homolgue as Las, can form intact flagellar basal bodies but had fewer and disoriented flagellar hooks and filaments (Zhang *et al.*, 2012). Accordingly, the motility of the *flgJ* mutant of *B. burgdorferi* was partially deficient. In addition, FlgJ seems to be functional because it was able to interact with FlgB. Thus, *Liberibacter* spp. may have evolved some novel means involving FlgJ to assemble the rod. Second, even though Las encodes a functional FlaA that complements the corresponding mutant of *A. tumefaciens*, the complex flagellar filament in *Rhizobiaceae* bacteria is formed by the interaction of FlaA with at least one secondary flagellin, such as FlaB, C or D, that is absent in Las. Ectopic expression of FlaA_Las_ in ΔflaABCD did not restore the motility of the null mutant, suggesting that FlaA_Las_ is incapable of forming a fully functional filament. Alternatively, the observation of flagella in *L. crescens* suggests synthesis of Las flagella related to FlgJ and FlaA might involve some novel mechanisms different from known examples in *Rhizobiaceae*. Lastly, even though we could not totally rule out the possibility that Las might contain flagella in certain conditions (such as in psyllids, as implied in Fig. 8), expression of the flagellar genes is repressed *in planta*. Indeed, western blotting was not able to detect flagellin *in planta*.

The flagellar features of Las *in planta* might help Las avoid the elicitation of plant defence responses since flagellin is known to trigger plant immunities as typical pathogen‐associated molecular patterns (Thomma *et al.*, 2011). Interestingly, mammalian cells use different receptors for recognition of extracellular flagellin or intracellular flagellin. For sensing flagellin outside the mammalian cell, the immune system uses Toll‐like receptor TLR‐5 (Yoon *et al.*, 2012). If flagellin protein is detected within the cytosol of a cell, it is detected by the Nod‐like receptor (NLR) Ipaf (Franchi *et al.*, 2006). We reason that flagellin might activate some early immune responses when Las is transmitted into the phloem by psyllids, during which the flagellin expressed in psyllids remain available *in planta*. It is likely the recognition is not mediated by FLS2 (Gómez‐Gómez and Boller, 2002), but instead by some unknown intracellular receptors. However, it is expected flagellar expression is turned off soon after transmission. Interestingly, multiple plant pathogenic bacteria, such as *Pseudomonas syringae* (Cheng *et al.*, 2018; Markel *et al.*, 2016) and *Xanthomonas axonopodis* pv. *glycines* (Chatnaparat *et al.*, 2016), have been known to use a similar strategy by reducing expression of flagellar genes and losing flagella once reaching their habitats inside the plant. On the other hand, the higher expression of flagellar genes in psyllids suggests flagella might be important for its movement inside the psyllid body to different organs and cell organelles (Ghanim *et al.*, 2017). The induction of flagellar genes in psyllids probably results from differential physiochemical conditions inside psyllids from the phloem sap. In addition, the ACP genome encodes a deficient innate immune system lacking a number of genes that encode for antimicrobial peptides and the Imd pathway associated with defence against Gram‐negative bacteria (Arp *et al.*, 2017).

**Figure 9:**
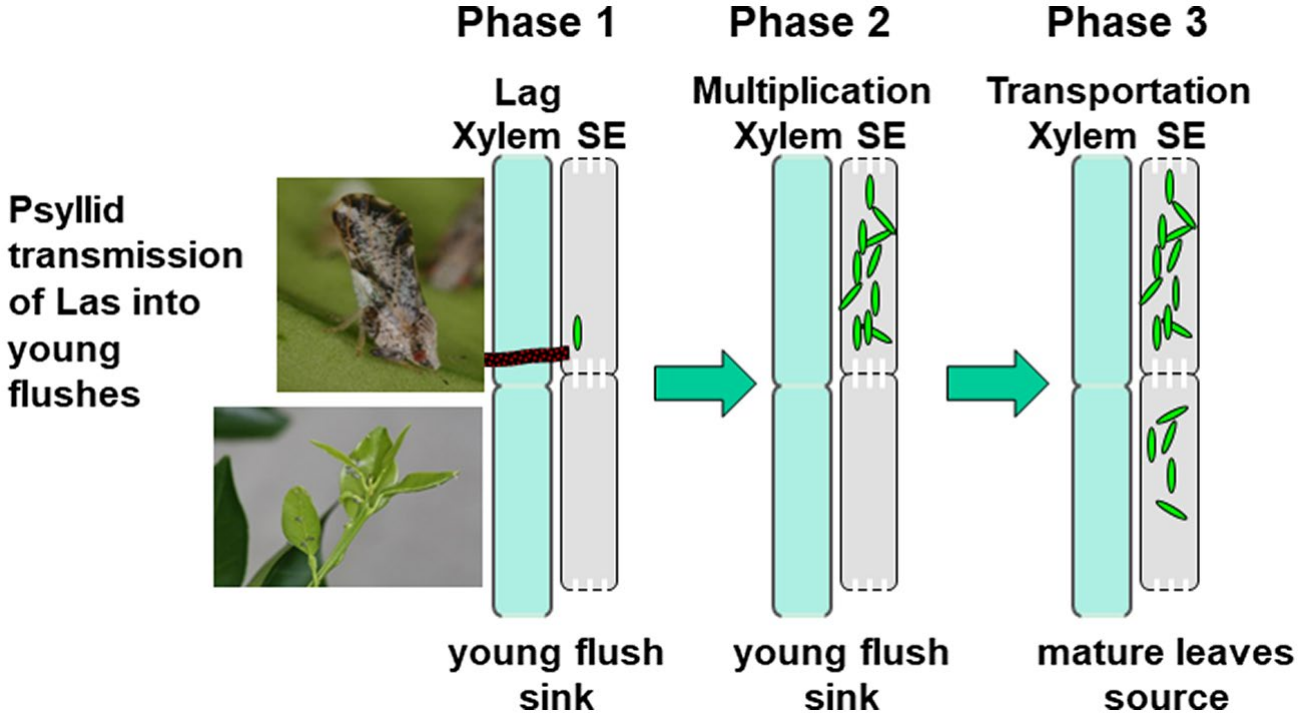
Schematic representation of ‘*Candidatus *Liberibacter asiaticus’ (Las) movement with phloem sap after psyllid transmission. Phase 1: After psyllid feeding and injecting Las into the phloem of young flush, Las initially undergoes a lag phase with no or slow growth to adapt to the environmental changes. Phase 2: Las begins multiplication inside the young flush after an undefined period. Phase 3: With the maturation of the young flush, the infected leaf transits from a sink into a source. Meanwhile, Las begins to move from the infected leaf to other parts of the plant, such as roots, following phloem sap from source to sink.

We have proposed the following movement model for Las after psyllid transmission into young flush (Fig. 9). In Phase 1 (lag phase), Las initially undergoes no or slow growth to adapt to the environmental changes from psyllids to phloem, then begins multiplication inside the young flush after an undefined period (Phase 2/multiplication phase). In the first two phases, Las stays inside the young flush before it turns into a source. With the maturation of the young flush, the infected leaf transits from a sink into a source. Meanwhile, Las begins to move from the infected leaf to other parts of the plant, such as roots, following phloem sap from source to sink (Phase 3/movement phase). The Las flagellar features probably suggest that Las moves with phloem sap rather than relying on flagella‐mediated movement. This model is consistent with the data that newly infected young leaves become infectious within 10–15 days after an inoculum of Las from an adult psyllid (Lee *et al.*, 2015). This movement pattern also promotes acquisition and transmission of Las by nymphs. Las has been suggested to move at approximately 2–3 cm/day in citrus after graft inoculation (S. Lopes, personal communication). However, we do not think that detecting Las in the leaves but not in the root results from the slow movement of Las. The average height (top to root) of the trimmed trees used for our Las movement assay is approximately 20 cm and we conducted the qPCR analysis at 54 days after psyllid feeding, which is supposed to be enough for Las to reach the root if moving right after feeding. In addition, microscopic analysis of Las *in planta* showed that Las cells float freely in the phloem sap without attaching to the sieve tube cell walls or each other (i.e. without forming biofilms) (Ding *et al.*, 2018; Folimonova and Achor, 2010; Hartung *et al.*, 2010; Hilf *et al.*, 2013; Kim *et al.*, 2009). A similar phenomenon has been observed for Lso and ‘*Ca*. Liberibacter americanus’ *in planta* (Liefting *et al.*, 2009; Secor *et al.*, 2009; Wulff *et al.*, 2014). The bacteria move primarily in the vertical direction along the sieve tubes through the sieve pores, rather than horizontally to adjacent sieve tubes. This hypothesis was first suggested by observations of the uneven colonization of Las in dodder, where adjacent phloem vessel elements were observed to be completely full of Las or free of the pathogen (Hartung *et al.*, 2010). Even though Las has delayed movement out of young flush, its population is highest in the seed coat compared to other tissues based on our experience. Dodder also hosts a high density of Las based on our previous experience. We used different tissues, including young flush, in our microscopy studies and could only observe Las when seed coats and dodder were used. Indeed, the procedure used to observe flagella under TEM is quite challenging for unculturable bacteria. More robust evidence for the Las movement model can be acquired by fluorescent microscopical analysis of green fluorescent protein (GFP)‐labelled Las, as previously conducted for xylem‐inhibiting *Xylella* (Newman *et al.*, 2003), provided Las can be cultured or be labelled by GFP *in vivo* in the future.

Those unique features of the HLB pathosystem centring on young flush suggest it represents the Achilles' heel for Las, and is thus the key point for HLB/ACP management. The psyllids preferentially feed and exclusively reproduce on young, newly emerged flush shoots of citrus, and their nymphs feed and complete their life stages on young flush shoots. Young flush is critical for psyllids acquiring and transmitting Las (Bové, 2006; Hall *et al.*, 2013; Setamou *et al.*, 2016; Sétamou and Bartels, 2015; Tomaseto *et al.*, 2016). The importance of controlling psyllids during flush stages has been recognized (Hall *et al.*, 2016). The delayed movement of Las out of the young flush provides opportunities to develop novel control strategies. Interestingly, traditional citrus growers in China not only rely on high‐frequency psyllid control of young flush using insecticides, but also remove flush (e.g. physical removal or spray with young flush killer) during summer or winter (for certain areas). This flush removal practice probably contributes to reducing the ACP population, preventing ACP from acquiring Las from young flush of HLB‐positive trees and transmitting Las to young flush. The delayed movement of Las in young flush also helps the development of targeted early detection of Las after psyllid transmission and before HLB symptom appearance (Pandey and Wang, 2019). In addition, targeted treatment of young flush with bactericides might prevent Las from establishing *in planta* after psyllid transmission. The minimum concentration of oxytetracycline required to suppress Las populations *in planta* has been suggested to be 0.68 and 0.86 µg/g under greenhouse and field conditions, respectively (Li *et al.*, 2019). Depending on application method, oxytetracycline concentrations in leaf tissues have been reported to range from 0 to 0.53 µg/g (Li *et al.*, 2019; Vincent *et al.*, 2019), below the concentrations required to suppress Las. It seems that successful prevention of Las establishment after psyllid transmission requires optimized delivery methods to increase bactericidal concentration to the minimum concentration of bactericides required to suppress Las populations *in planta*. The bactericidal effect needs to cover the whole flush period until leaf maturation. Additionally, it is interesting to know whether the targeted treatment effect can be obtained with non‐antibiotic bactericides such as generally recognized as safe (GRAS) antimicrobial compounds.

In summary, the flagellar characteristics of Las support a delayed movement of Las from young flush after psyllid transmission. The movement pattern of Las provides unique opportunities for early diagnosis of HLB and targeted treatment of young flush to control HLB.

## Experimental Procedures

### Bacterial strains, plasmids and growth conditions

The plasmids, oligonucleotides and bacterial strains used in this study are listed in Tables S1 and S2. *Escherichia coli* cells were grown at 37 °C in Luria–Bertani (LB) medium. The *A*. *tumefaciens* strain C58 and mutant strains were grown in LB medium at 28 °C. Plasmids were introduced into *E. coli* by heat‐shock at 42 °C and into *A*. *tumefaciens* by electroporation. *A* *grobacterium* *tumefaciens* deletion mutants were generated using the suicide vectors pNPTS138 as described elsewhere (Hibbing and Fuqua, 2011). Cells of *L. crescens* strain BT‐1 were grown at 28 °C at 150 rpm in BM7 medium. Antibiotics were used at the following concentrations: kanamycin 50 mg/mL, ampicillin 100 mg/mL, gentamicin 10 mg/mL.

### Deletion of flagellar genes and construction of complemented strains of *A. tumefaciens*

DNA manipulations and PCR were performed according to standard procedures (Sambrook *et al.*, 2006). To construct the *flaA, flaD* and *flgJ* deletion mutants, approximately 1 kb of the upstream and downstream regions of those flagellar genes were amplified by PCR from *A. tumefaciens* genomic DNA (oligonucleotides are listed in Table S2), and the two fragments were ligated to produce an in‐frame deletion. The approximately 2 kb fragments that resulted were then cloned into the *Hin*dIII site of the pNPTS138 suicide vector, generating the plasmids pNPTS‐FlaA, pNPTS‐FlaD and pNPTS‐FlgJ (Andrade and Wang, 2019; Andrade *et al.*, 2014). These constructs were introduced into *A. tumefaciens* cells by electroporation, and the wild‐type copies were replaced by the deleted version after two recombination events. For the first and second recombination events, *A. tumefaciens* cells were selected in LB medium without NaCl (LBON) with kanamycin and in LBON plus 5% sucrose, respectively. The single mutants ∆*flaA*, ∆*flaD* and ∆*flgJ* and the double mutant ∆*flaAD* were confirmed by PCR. To produce the flagellin null mutant, two fragments of 1 kb of the upstream and downstream regions of *flaA* and *flaC* were amplified and ligated. A fragment of 2 kb was generated and cloned into the *Hin*dIII site of pNPTS138. This resulting construct pNTPS‐FlaABC, carrying in‐frame deletion of *flaA‐flaB‐flaC*, was introduced into *A. tumefaciens flaD* mutant strain by electroporation. After selections in appropriate media, the null mutant ∆*flaABCD* was confirmed by PCR. To complement ∆*flgJ*, ∆*flaA*, the double mutant ∆*flaAD* and the null mutant strains, fragments including the coding region of *flaA* or *flgJ* genes were amplified by PCR from Las genomic DNA. Also, the coding region of *flgJ* was amplified from *A*. *tumefaciens* genome. Those three fragments were inserted into the *Bam*HI site of the pTF53 vector that contains the constitutive promoter Trp (Andrade and Wang, 2019; De Feyter *et al.*, 1993), creating the plasmids pF53‐Las_FlaA, pF53‐Las_FlgJ and pF53‐Atu_FlgJ. The construct pF53‐FlaA_Las was used to transform ∆*flaA*, ∆*flaAD* and ∆*flaA* *BCD* mutant strains. ∆*flgJ* mutant strain was transformed with pF53‐Las_FlgJ or pF53‐Atu_FlgJ constructs. After electroporation, the transformed *A*. *tumefaciens* cells were selected on LB medium with gentamicin. All constructs were sequenced for confirmation.

### Motility assay

The swimming motility of *A. tumefaciens* was assayed on semisolid agar as described previously (Merritt *et al.*, 2007). Briefly, bacteria cells grown in LB media for 48 h at 28 °C were stabbed in 0.3% (w/v) agar ATGN modified medium plates (20 mM NaCl, 10 mM (NH_4_)_2_SO_4_, 5 mM MgSO_4_, 1 mM CaCl_2_, 0.16 mM KH_2_PO_4_, 0.32 mM K_2_HPO_4_, 0.01 mM FeSO_4_, 10 mM fructose, 10 mM sucrose, 0.03% casamino acid). Motility was evaluated and photographed after 3 days of incubation at 28 °C. The means of three replicates and standard deviation values were calculated. Statistical analysis was conducted by Student’s *t*‐test (*P* < 0.05).

### Pull‐down assay

The constructs used in the pull‐down assay are listed in Table S1. Those constructs were used to transform *E. coli* strain BL21(DE3)*.* The transformants were precultured overnight in 3 mL of LB medium plus specific antibiotics (Table S1) at 37 °C and 200 rpm. Then, precultured cells were diluted 1:100 in 100 mL of LB medium and grown at 37 °C until OD_600nm_ = 0.6. The expression of the recombinant proteins was induced by addition of 1 mM IPTG to the growth medium and the induction process occurred at 30 °C and 200 rpm for 6 h. After induction, *E. coli* cells were pelleted by centrifugation at 2348 *g* and 4 °C for 10 min. Pelleted *E. coli* cells expressing GST‐FlgJ_Las_ protein were washed in phosphate‐buffered saline (PBS, pH 7.4) and suspended into lysis buffer (PBS, 100 µg/mL lysozyme and 40 µg/mL DNAse I) and sonicated to generate the cell lysates. After centrifugation, the cell lysates were incubated with 0.3 mL of glutathione sepharose high‐performance beads for 1 h then beads mixed with cell lysates were loaded on a 10 mL column, according to the manufacturer’s instructions (GE Healthcare, Pittsburgh, PA, USA). The beads were washed four times with PBS to remove the unbound proteins and incubated with *E. coli* cell lysates containing 6×HisEGFP, 6×HisFLgB or 6×HisFliE for 2 h at 4 °C. After washing three times, the proteins were eluted with PBS plus reduced glutathione (7 mg/mL). Eluted samples were mixed with 4× SDS‐PAGE loading buffer and boiled for 5 min. The controls (Inputs) and eluted samples were subjected to SDS 12% polyacrylamide gel electrophoresis and immunoblotting by using anti‐GST (1:5000) and anti‐6×His (1:3000) (Sigma, St Louis, MO, USA) antibodies followed by secondary antibodies raised against rabbit (1:10 000) (Sigma).

### Las‐infected psyllids and plants

Las‐infected psyllid colonies were reared in laboratory cultures maintained on Las‐infected sweet orange trees at the University of Florida, Citrus Research and Education Center (CREC‐UF, Lake Alfred, FL, USA). Psyllid colonies were maintained in insect‐proof cages inside environmentally controlled growing chambers with a 12‐h photoperiod. Las‐infected seeds were obtained from lopsided fruits from HLB‐positive grapefruit (*Citrus paradisi*). Las‐infected dodder plants (*Cuscuta* sp.) on HLB‐positive periwinkle (*Catharanthus roseus*) were used for TEM assay. Plants were grown in a greenhouse with a 12‐h photoperiod and controlled temperature. Las‐positive dodder tendrils were a gift from Dr Ed Etxeberria (University of Florida, CREC, Lake Alfred, FL, USA). Las in the insects, citrus plants, seed coats and dodder plants was verified by qPCR (Hartung *et al.*, 2010; Hilf *et al.*, 2013; Yan *et al.*, 2013). A sleeve net (80 mesh) was used to cage Las‐positive psyllids on young flush only. The caged plants were kept within a bigger cage to prevent interference from other psyllids.

### Isolation of Las from psyllid guts and plant materials

About 80 Las‐infected adult psyllids (Ct value = 26 to 32) were collected from the psyllid room (CREC‐UF) and used to dissect midguts in sterile PBS under a dissecting stereomicroscope by using depressed glass wells and fine entomological needles. After tissue samples were dissected, the tissues were washed three times with PBS and transferred to a sterile Eppendorf tube containing 0.3% bacitracin in PBS (Ghanim *et al.*, 2016). Samples were pipetted up and down slowly to release the bacteria cells attached to the midgut.

Selected seeds isolated from infected and symptomatic citrus plants (Ct value about 27 to 29) were washed five times in sterile water, dried in filter paper and the coats were removed by using high‐precision straight fine tweezers. Seed coats were divided into very small pieces and suspended with 0.3% bacitracin (Thermo Fisher, Waltham, MA, USA) in PBS.

Individual dodder tendrils positive for Las (Ct = 24 to 26) were removed intact from parasitized plants. A segment of 10 mm was removed from the centre of dodder stem pieces, washed five times in sterile water, dried in paper filter, transferred to 0.3% bacitracin in PBS and cut into very small pieces with razor blades. The mixtures were pipetted up and down slowly to release the bacteria. Solution containing Las was transferred to a sterile Eppendorf tube.

### Transmission electron microscopy and antibody‐labelled Las cells

Bacterial cultures of *L. crescens* were grown at 28 ºC in BM7 medium with 5 mM putrescine (Sigma) to OD_600nm_ of 0.5. One droplet of suspended bacteria was placed on formvar‐ and carbon‐coated 400‐mesh copper grids and allowed to settle for 1 min. The excess water was wicked away with filter paper. The samples were then stained, before drying, with 1% aqueous ammonium molybdate (Sigma) to which a few drops of bacitracin water (20 μg of bacitracin dissolved in 10 mL water) was added as a spreading agent. The stains were added to the grids and wicked away with filter paper immediately. The grids were allowed to dry for 1 h before being viewed and photographed on a Morgagni 268 transmission electron microscope (FEI Company, Eindhoven,  Netherlands). Similar procedures were repeated for bacterial suspension diluted 1:5 in PBS obtained from Las‐infected psyllid midguts, grapefruit seed coats and dodder stems.

The labelling of Las bacteria absorbed in the grids was done as follows. Grids with absorbed Las cells were incubated with polyclonal antibody against Las anti‐OmpA raised in rabbit (Abcam, Cambridge, MA, USA). Rabbit antisera against Las OmpA was diluted 1:250 in PBST buffer [1x PBS, 1% (w/v) bovine serum albumen and 1% (v/v) Triton X‐100]. After 1 h incubation, the grids were washed three times for 5 min each in PBST buffer. Grids were incubated with anti‐rabbit IgG (whole molecule)‐gold (Sigma) diluted 1:5 in PBST buffer and incubated for 30 min. After incubation, the grids were washed three times in PBST buffer and twice in sterile water. Finally, grids were stained with 1% aqueous ammonium molybdate and after drying for 1 h they were visualized and photographed on a Morgagni 268 TEM (FEI Company).

### Flagellin antibody and detection of flagellin via western blotting

To detect the flagellin protein in different samples, the peptide DRVSSGLRVSDAAD of Las flagellin was synthesized as an antigen to produce Las flagellin antibody from rabbits. The total proteins were isolated from *A. tumefaciens *strain GV2260, Las‐infected citrus and Las‐infected psyllids. *Agrobacterium tumefaciens* cells were grown in YEP medium to OD_600 nm_ = 0.6. Cells were pelleted by centrifugation, resuspended in 2× SDS‐PAGE loading buffer and boiled for 5 min. Protein extracts from both Las‐infected and Las‐free psyllids (ten insects) and citrus plants (pieces of petioles) were obtained by snap freezing the samples in liquid nitrogen following maceration of the tissues in tissue Lyser (Qiagen, Germantown, MD, USA). Macerated psyllid samples were resuspended in I‐PER Insect Cell protein extraction reagent (Thermo Fisher), and macerated citrus samples were resuspended in CelLytic P Cell lysis reagent (Sigma). Resuspended samples were centrifuged at 13 523 *g* for 15 min at 4 °C, then the supernatants were diluted in 2× SDS‐PAGE loading buffer. Ten microlitres of protein of each sample was loaded for 10% SDS‐PAGE electrophoresis and western blot analysis. Full‐length flagellin fused with a maltose‐binding protein (MBP) tag was used as a positive control. Las‐free citrus and psyllids, and free MBP protein were used as negative controls.

### RT‐qPCR analysis of Las flagellar genes

Comparative analysis of expression of the selected flagellum genes encoded by the Las genome was verified in Las‐infected plants of Valencia sweet orange (*C. sinensis*) and in Las‐infected psyllids as described previously (Yan *et al.*, 2013). All RT‐qPCRs were performed using a 7500 Fast Real‐time PCR system (Applied Biosystems, Foster City, CA, USA) with a QuantiTect SYBR Green RT‐PCR kit (Qiagen). The primers were designed from the sequence of the Las genome using IDT SciTolls (http://www.idtdna.com/pages/scitools). The total reaction volume of one‐step RT‐qPCR was 25 μL and contained 2 × QuantiTect SYBR Green RT‐PCR Master Mix (12.5 μL), 10 μM gene‐specific primers (1.25 μL), QuantiTect RT Mix (0.5 μL) and 50 ng of RNA template (1 μL). 16S rRNA was used as the endogenous control. Reactions were incubated at 50 °C for 30 min and at 95 °C for 15 min, then cycled (40 times) at 94 °C for 15 s, 54–56 °C for 30 s and 72 °C for 30 s. Melting curve analysis was conducted to verify the specificity of the RT‐qPCR products. Two technical replicates and three biological replicates were used for each of the genes. The relative fold change in target gene expression was calculated using the formula 2^−^^ΔΔCt^ (Livak and Schmittgen, 2001). Statistical analysis of all data was conducted by Student’s *t*‐test.

## Supporting information


**Fig. S1** Sequence alignment of the peptidoglycan hydrolase encoded by ‘*Candidatus* Liberibacter’ species was generated by ClustalW. The signal peptide sequence found by PrediSi is highlighted in yellow. The SLT domain is indicated in green. CLas, ‘*Ca. *Liberibacter asiaticus’ (CLIBASIA_00965); CLaf, ‘*Ca.* Liberibacter africanus’ (G293_01215); CLam, ‘*Ca.* Liberibacter americanus’ (Lam_376); CLso, ‘*Ca.* Liberibacter solanacearum’ (CKC_02595); Lcc, *Liberibacter crescens* BT‐1 (B488_10490).

**Fig. S2** Swimming motility assay of the *Agrobacterium tumefaciens* wild‐type, *ΔflaAD* and *ΔflaABCD* mutant strains, and the mutant strains complemented with ‘Candidatus Liberibacter asiaticus’ (Las) *flaA*. The mean values ± the standard deviations (*n* = 3) are plotted. Mean values were compared to the wild‐type, * indicate statistically significant difference (*P* < 0.05, Student *t* test). WT, *A. tumefaciens* wild‐type strain carrying the empty vector. *ΔflaAD* and *ΔflaABCD*, *A. tumefaciens* mutant strains carrying the empty vector. *ΔflaAD* + *flaALas* and *ΔflaABCD* + *flaALas*, mutant strains carrying the Las *flaA* gene.

**Table S1** Bacterial strains and plasmids used in this study.

**Table S2** Oligonucleotides used in this study.

## Data Availability

The data that support the findings of this study are available from the corresponding author on reasonable request.
