# Supplementary material for: The flagella of ‘Candidatus Liberibacter asiaticus’ and its movement in planta
Source: Mol Plant Pathol. 2019 Nov 13;21(1):109–23. doi: 10.1111/mpp.12884 (PMC6913195; doi:10.1111/mpp.12884)
Supplement: Supplementary file 1 — Fig. S1 Sequence alignment of the peptidoglycan hydrolase encoded by ‘Candidatus Liberibacter’ species was generated by ClustalW. The signal peptide sequence found by PrediSi is highlighted in yellow. The SLT domain is indicated in green. CLas, ‘Ca. Liberibacter asiaticus’ (CLIBASIA_00965); CLaf, ‘Ca. Liberibacter africanus’ (G293_01215); CLam, ‘Ca. Liberibacter americanus’ (Lam_376); CLso, ‘Ca. Liberibacter solanacearum’ (CKC_02595); Lcc, Liberibacter crescens BT‐1 (B488_10490). [file MPP-21-109-s001.docx]

**Lcc MLFKNPKLLIISSIIAVVLPELALA------------------------------CGNTS 30**

**CLam MFSKRFIIQKIVLLIALYFVSTMTICSDNLDSFKEWISEKKGINRKVSPVSTVSICSDNP 60**

**CLas MSFKKY—LLISLLMMVYPM-LAQA-------------------VTQPKKATTQFICSDTA 39**

**CLaf MSFREIKSLFLLFTILMYPTLSQA------------------------------ICSESP 30**

**CLso MSFNKTRSLFILLLILIHPTLSLA------------------------------ICSETP 30**

*** .. : : : *.:.**

**Lcc DGFKKWLSNAHTDAINKGIDQEILNKIFPTISYNLATINADRNQS-SLKLSFDDFLQKRG 89**

**CLam DNFKEWISEARNEAIKKGINIKIVNDIFSNLEYSFKTIELDRKQT-IFKLSFDEFLKKVS 119**

**CLas DRFGEWKSAARARAVREGMSPKTAKHLFADLEYLDTTIARDRKMVTSPPVSFKDYLDGLS 99**

**CLaf DRFNEWISDTREQALKNGVHKEIADEVFSNLQYREATINADRKMFSSPPLSFKEYLETIP 90**

**CLso DKFNEWMSDAREQALKKGINADVVNKVFSNIKYRDATVSLDRKMFTAPPLSFQEYISGIA 90**

*** * :* * :: *:.:*: . ..:* :.* *: **: :**.:::.**

**Lcc VNNIVNKGKVLKKKYANLFKSIKEIYGVPAGPLLAIWGMETAFGTSIGKQHTLSALATLA 149**

**CLam AASVIENGKSMKRKYSDFLKKVEKNYGVSPGVLMALWGLESKFGTKKGTINTLSALATLS 179**

**CLas SSEIIAQGIALKKANSRLLINLKKEYGVPPGILMALWGLETHFGETMGKTPLLSTLATLA 159**

**CLaf YSTIIATGKQLKKKNEEAFRHLKRKYGVPAGVIISLWGLESSFGTRMGKVPILSTLATLA 150**

**CLso TSNIIERGEKIKKEYQALFQKIQKDYGVPAGILITLWGLESRFGDRMGEVPTFSTLATLA 150**

**:: * :*: : ::. *** * ::::**:*: ** * :*:****:**

**Lcc YDCRRSAFFTEQFNIALELIAEGKMDPNSRGALHGEIGQFQFLPSNLKKFGVDGNNDNVV 209**

**CLam YDCRRSKLFTEQFFTAVSLVNNGTISIDSIGAPHGEIGQFQFLPTNVERFAVDADGDGKA 239**

**CLas YDCRRAKFFTEQFFYALDLVKKGVISSHALGATFGEIGQFQFLPVNVVKYAIDADEDGKI 219**

**CLaf YDCRRSKFFTEEFFAALYLVDKNIISPTSLGAMYGEIGQFQFLPSNVKKFAVDADEDGQA 210**

**CLso YDCRRSALFTEQFFVALELVNQGIISAESRGALHGEIGPFQFLPSNVKKFSVDGDGDGKA 210**

*******: :***:* *: *: :. :. : ** .**** ***** *: ::.:*.: *.**

**Lcc DIINSKADAFASTANFFKKNGWQPGVGYQPDEPNFKAIKSWNASTVYQKAIAYAAAKIDE 269**

**CLam DVVNSNLDAIESAANFLKKSGWNAMYGYQPQEPNFKILNRWNASMVYKKTVAYIAANIDG 299**

**CLas DLVKSNIDAIASAAKFLAKLGWTKCAGYQPGEKNFAILKHWNASLNYNKTIAYIAAHIDG 279**

**CLaf NLIESPTDAIVSAANFLSKNGWVRCKGYQPTEANFATLKRWNSSTNYIKTVAYIAAHIDG 270**

**CLso SIITSNIDAIESAANFLKKNGWIKNKGYQPEEKNFLILKRWNNSTNYIKAVAYIAAHIDG 270**

**.:: * **: *:*:*: * ** **** * ** :: ** * * *::** **:****

**Lcc IKLKKVY-- 276**

**CLam LKIKDVYE- 307**

**CLas VPIGKGYDT 288**

**CLaf IKLNRKYD- 278**

**CLso IKLKDGYQ- 278**

**: : ***

**Figure S1.** Sequence alignment of the peptidoglycan hydrolase encoded by *Ca*. Liberibacter species was generated by ClustalW. The signal peptide sequence found by PrediSi is highlighted in yellow. The SLT (Soluble Lytic Transglycosylase) domain is indicated in green. CLas, *Ca.* Liberibacter asiaticus (CLIBASIA_00965); CLaf, *Ca.* Liberibacter africanus (G293_01215); CLam, *Ca.* Liberibacter americanus (Lam_376); CLso, *Ca.* Liberibacter solanacearum (CKC_02595); Lcc, *Liberibacter crescens* BT-1 (B488_10490).
