# Supplementary material for: The flagella of ‘Candidatus Liberibacter asiaticus’ and its movement in planta
Source: Mol Plant Pathol. 2019 Nov 13;21(1):109–23. doi: 10.1111/mpp.12884 (PMC6913195; doi:10.1111/mpp.12884)
Supplement: Supplementary file 2 — Fig. S2 Swimming motility assay of the Agrobacterium tumefaciens wild‐type, ΔflaAD and ΔflaABCD mutant strains, and the mutant strains complemented with ‘Candidatus Liberibacter asiaticus’ (Las) flaA. The mean values ± the standard deviations (n = 3) are plotted. Mean values were compared to the wild‐type, * indicate statistically significant difference (P < 0.05, Student t test). WT, A. tumefaciens wild‐type strain carrying the empty vector. ΔflaAD and ΔflaABCD, A. tumefaciens mutant strains carrying the empty vector. ΔflaAD + flaALas and ΔflaABCD + flaALas, mutant strains carrying the Las flaA gene. [file MPP-21-109-s002.pptx]

## Slide 1
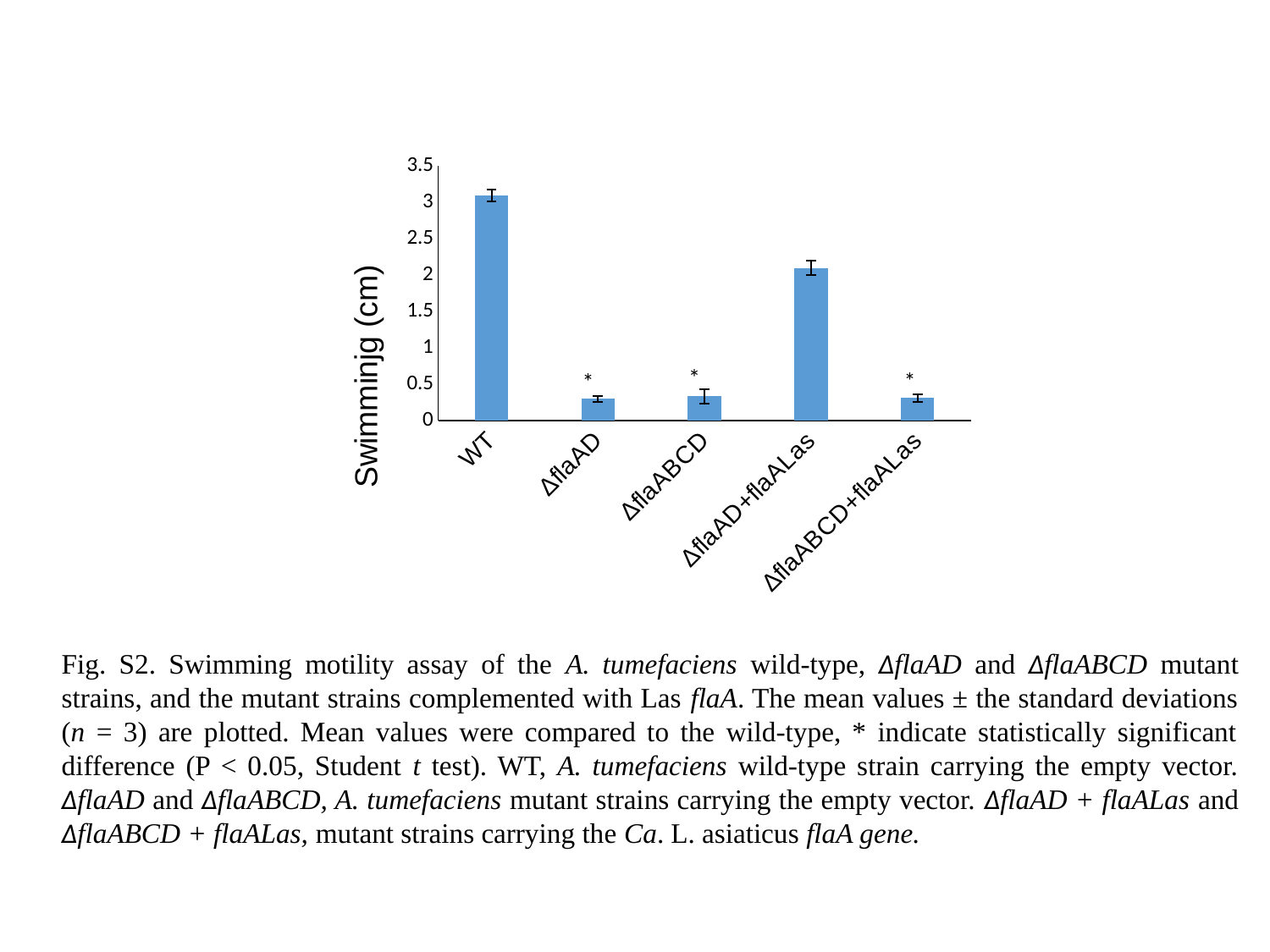

### Chart
| Category | Aveg |
|---|---|
| WT | 3.1 |
| ∆flaAD | 0.3 |
| ∆flaABCD | 0.3333333333333333 |
| ∆flaAD+flaALas | 2.1 |
| ∆flaABCD+flaALas | 0.3133333333333333 |*
*
*
Fig. S2. Swimming motility assay of the A. tumefaciens wild-type, ∆flaAD and ∆flaABCD mutant strains, and the mutant strains complemented with Las flaA. The mean values ± the standard deviations (n = 3) are plotted. Mean values were compared to the wild-type, * indicate statistically significant difference (P < 0.05, Student t test). WT, A. tumefaciens wild-type strain carrying the empty vector. ∆flaAD and ∆flaABCD, A. tumefaciens mutant strains carrying the empty vector. ∆flaAD + flaALas and ∆flaABCD + flaALas, mutant strains carrying the Ca. L. asiaticus flaA gene.
