# Supplementary material for: The flagella of ‘Candidatus Liberibacter asiaticus’ and its movement in planta
Source: Mol Plant Pathol. 2019 Nov 13;21(1):109–23. doi: 10.1111/mpp.12884 (PMC6913195; doi:10.1111/mpp.12884)
Supplement: Supplementary file 3 — Table S1 Bacterial strains and plasmids used in this study. [file MPP-21-109-s003.docx]

**Table S1. Bacterial strains and plasmids used in this study.**

| **Strain or plasmid** | **Relevant characteristics** | **Reference** |
| --- | --- | --- |
| **Strains** |  |  |
| ***Escherichia coli*** |  |  |
| **DH5α** | F^–^ *rec*A (r_K_–, m_K_+) *hsd*R17 (rΔ(*lac*ZYA-*arg*F) Φ80d*lac*Z ΔM15 | New England Biolabs (Ipswich, MA, USA) |
| **BL21 Star (DE3)** | F^–^  ompT hsdSB (r_B_- m_B_-) gal dcm rne131 (DE3) pLysS (Cam^r^) | Life Technologies (Carlsbad, CA, USA) |
| ***Agrobacterium tumefasciens* (Atu)** |  |  |
| **C58** | Wild type | Gift from Dr. Stanton Galvin, Purdue University |
| ***ΔflaAD*** | In frame deletions of *flaA and flaD* | This study |
| **Δ*flaABCD*** | *flagellin null mutant* | This study |
| ***ΔflgJ*** | In frame deletions of *flaJ* | This study |
| ***ΔflaAD-p53*** | Empty pUFR053 plasmid in the *ΔflaAD* | This study |
| ***ΔflaABCD-p53*** | Empty pUFR053 plasmid in the *ΔflaABCD* | This study |
| ***ΔflgJ-p53*** | Empty pUFR053 plasmid in the *ΔflgJ* | This study |
| ***ΔflaAD* -CLas_flaA** | *ΔflaAD* strain carrying the construct *p53-CLas_flaA* | This study |
| ***ΔflaABCD* -CLas_flaA** | *ΔflaABCD* strain carrying the construct *p53-CLas_flaA* | This study |
| ***ΔflgJ-CLas_*flgJ** | *ΔflaAD* strain carrying the construct *p53-CLas_flgJ* | This study |
| ***ΔflgJ-Atu_*flgJ** | *ΔflgJ* strain carrying the construct *p53-CLas_flgJ* | This study |
| ***Liberibacter crescens*** |  |  |
| ***BT-1*** | Wild type | Gift from Dr. Mike Davis, CREC-UF |
| ***Candidatus Liberibacter asiaticus*** | Wild type | Isolated from psyllid midguts and host plants |
| **Plasmids** |  |  |
| **pTF53** | pUFR053 derivative containing the *trp* promoter | (Andrade and Wang, 2019) |
| **p53-CLas_flaA** | Coding region of *hrpG* cloned in pUFR053 | This study |
| **p53-CLas_flgJ** | Coding region of *hrpG* cloned in pUFR053 | This study |
| **p53-Atu_flgJ** | Coding region of *hrpG* cloned in pUFR053 | This study |
| **pNTPS138** | Suicide vector for generation of gene knockouts, *sacB* and Km^r^ | (Andrade et al., 2014) |
| **pNPTS-flaA** | pNPTS138 derivative for generation of *flaA* knockout, *sacB* and Km^r^ | This study |
| **pNPTS-flaD** | pNPTS138 derivative for generation of *flaD* knockout, *sacB* and Km^r^ | This study |
| **pNPTS-flaABC** | pNPTS138 derivative for generation of *flaABC* knockout, *sacB* and Km^r^ | This study |
|  |  |  |
| **pET28a(+)** | *E. coli* expression vector, Km^r^ | Novagen (Madison, WI, USA) |
| **pET-6HisFlgB** | CLas *flgB* cloned into pET28a*_Nde_*_I/_*_Hind_*_III_ | This study |
| **pET-6HisFliE** | CLas *fliE* cloned into pET28a*_Nde_*_I/_*_Hind_*_III_ | This study |
| **pGEX-4TI** | *E. coli* expression vector, Ap^r^ | GE Healthcare (Pittsburgh, PA, USA) |
| **pGEX-FlgJ_Las_** | CLas *flgJ* cloned into pGEX-4TI*_BamHI_*_/_*_XhoI_* | This study |
| **pETHis6-EGFP** | EGFP cloned into pET28a (Addgene #29663) | Gift from Dr. Scott Gradia |

Gm^r^, gentamycin-resistant; Km^r^, kanamycin-resistant; Cm^r^, chloramphenicol-resistant; Ap^r^, ampicillin-resistant.
