# Supplementary material for: The flagella of ‘Candidatus Liberibacter asiaticus’ and its movement in planta
Source: Mol Plant Pathol. 2019 Nov 13;21(1):109–23. doi: 10.1111/mpp.12884 (PMC6913195; doi:10.1111/mpp.12884)
Supplement: Supplementary file 4 — Table S2 Oligonucleotides used in this study. [file MPP-21-109-s004.docx]

**Table S2. Oligonucleotides used in this study.**

| **Name** | **Sequence (5’ to 3’)** | **Cloning sites** |
| --- | --- | --- |
| ***For mutant construction*** |  |  |
| **F1ΔflaA** | GGCTGGCGCCAAGCTTCATCCTGATCATGGTCACGAGCT | *HindIII* |
| **R1ΔflaA** | GTTGAATTCGTTGTTGGTCAGAATGCTTGCCAT |  |
| **F2ΔflaA** | AACAACgaattcAACAGCGACTCGCAGAACATCCTG |  |
| **R2ΔflaA** | TCCTGCAGAGAAGCTTCGTATTTCGTGGTCGTAACGCTAA |  |
| **F1ΔflaD** | GGCTGGCGCCAAGCTTGCCAGGACCTTATCGCATTGAA | *HindIII* |
| **R1ΔflaD** | GATGAATTCGAGGTTCTTGTCGATCATACGCAG |  |
| **F2ΔflaD** | AACCTCgaattcATCCTCCAGCTCTTCAAGTAACGG |  |
| **R2ΔflaD** | TCCTGCAGAGAAGCTTCTTGTGGCGCTTGACGATGATGA |  |
| **F1ΔflaABC** | GGCTGGCGCCAAGCTTCATCCTGATCATGGTCACGAGCT | *HindIII* |
| **R1ΔflaABC** | GTTGAATTCGTTGTTGGTCAGAATGCTTGCCAT |  |
| **F2ΔflaABC** | CAACAACgaattcAATATCCTGTCGCTATTCCGCCAGTAA |  |
| **R2ΔflaABC** | TCCTGCAGAGAAGCTTTATCGACGCCATGATTGCCGAGAATG |  |
| **F1ΔflgJ** | GGCTGGCGCCAAGCTTCCGATCCCATCGAAGACGTGCA | *HindIII* |
| **R1ΔflgJ** | TTCTCAGAATTCCGCCGCACGAACCACATCCAT |  |
| **F2ΔflgJ** | GCGGAATTCtgagaaTGAAAAACCTTAACGAGGAGAC |  |
| **R2ΔflaJ** | TCCTGCAGAGAAGCTTTCGTGAATGGAGCAAGAGATGATTAAC |  |
| ***For complementation*** |  |  |
| **Clas_flaA-F** | AGGAAAAACATATGGGTACCATGACTAGTATTTTAACCAATCACTCTGCA | *BamHI* |
| **CLas_flaA-R** | AGGAAGGATCCTGCGGTACCAGCACTACCATTGAGTGAATTTTAACCAC |  |
| **Atu_flaA-F** | AGGAAAAACATATGGGTACCATGGCAAGCATTCTGACCAACAACA | *BamHI* |
| **Atu_flaA-R** | AGGAAGGATCCTGCGGTACCTTAGCGGAAGAGCGACAGGATGTTCT |  |
| **Atu_flgJ-F** | AGGAAAAACATATGGGTACCatggatgtggttcgtgcggcg | *BamHI* |
| **Atu_flgJ-R** | AGGAAGGATCCTGCGGTACCTCAAGCCTCGTCCGTTTTATCATTGGA |  |
| **CLas_flgJ-F** | AGGAAAAACATATGGGTACCATGGATGTGGTTCGTGCGGCGGA | *BamHI* |
| **CLas_flgJ-R** | AGGAAGGATCCTGCGGTACCTCAAGCCTCGTCCGTTTTATCATTGGAA |  |
| ***CLas diagnostic*** |  |  |
| **CQULA04F** | TGGAGGTGTAAAAGTTGCCAAA |  |
| **CQULA04R** | CCAACGAAAAGATCAGATATTCCTCTA |  |
| ***For qRT-PCR*** |  |  |
| ***fliG*-F** | AGGCTTGGAACAAAATGAGTTG |  |
| ***fliG* -R** | AGCAGTGGTTTGAGGATGTTC |  |
| ***fliQ*-F** | AGAAGTAACTCTGACCTTTGTTCC |  |
| ***fli*Q-R** | AATACGTGAAAGCACCAAAGTG |  |
| ***flgK*-_-_F** | ATGGCGAGGGTTCTTATTGG |  |
| ***flgK*-R** | AATGGTTTCAGAGATCCCCTG |  |
| ***flhA*-F** | ACAACGGATATCTCTTTGCCTG |  |
| ***flhA*-R** | GTTCTTTGACCTCATCTCCTGG |  |
| ***flgE*-F** | GCTACTGTTCCAAGCGAAGA |  |
| ***flgE*-R** | CAACATTGGCAGTTTCTAGCG |  |
| ***flgL*-F** | CGAAGAGATGGTTACAGGACAG |  |
| ***flgL*-R** | CGAGACTGTTAGAATGGAGCC |  |
| ***flaA*-F** | ACTCAGGCTATTCTATGGTAACAC |  |
| ***flaA*-R** | TTGATCCTATTTTCCCCGCC |  |
| ***fliP*-F** | TTTACTATTTTGTCAATCGCTCCG |  |
| ***fliP*-R** | GTGCAAGGCTGATTATAACAAGG |  |
| ***fliL*-F** | ATAGCGGATTCTGTTCGTAGC |  |
| ***fliL*-R** | ATCAGCACTCCAAGCCTTATC |  |
| ***flgA*-F** | TCGAATCATTCTCACCCAAGG |  |
| ***flgA*-R** | ACTTCCAGAAACCATGACCC |  |
| **16S-F CLIBASIA_r05781** | GGATAACGCATGGAAACGTGTGCT |  |
| **16S-R CLIBASIA_r05781** | AATCCAACGCAGGCTCATCTCTCT |  |
| ***For expression in E. coli*** |  |  |
| **CLas flgJ-F** | TGGTTCCGCGTGGATCCatgcaagtccttcctattagtaatatc | *BamHI* and *XhoI* |
| **CLas flgJ-R** | CGATGCGGCCGCTCGAGTCACTTACTTATCTCAGGTAAATCAAG |  |
| **CLas flgB-F** | CGCGCGGCAGCCATATGCAACCGATTACATTTTTTCAAATTG | *NdeI* and *HindIII* |
| **CLas flgB-R** | AGTGCGGCCGCAAGCTTATCCCCTTACCACATGCATCACCA |  |
| **CLas fliE-F** | CGCGCGGCAGCCATATGattgaacaaattcaaggcacaaatag | *NdeI* and *HindIII* |
| **CLas fliE-R** | AGTGCGGCCGCAAGCTTCATATCTGCATTTTAGAAACTTCCG |  |
